# High parental monitoring prevents adolescents from engaging in risky sexual practices in Harar, Ethiopia

**DOI:** 10.3402/gha.v7.25724

**Published:** 2014-11-12

**Authors:** Yadeta Dessie, Yemane Berhane, Alemayehu Worku

**Affiliations:** 1 College of Health and Medical Sciences, Haramaya University, Harar, Ethiopia; 2 Addis Continental Institute of Public Health, Addis Ababa, Ethiopia; 3 School of Public Health, Addis Ababa University, Addis Ababa, Ethiopia

**Keywords:** risky sexual practices, adolescents, parent, communication, monitoring, Harar, Ethiopia

## Abstract

**Background:**

Emerging findings have shown that high parental monitoring of adolescents’ sexual and reproductive health (SRH) communications between parents and adolescents and good parenting styles prevent adolescents from engaging in risky sexual practices.

**Objective:**

The aim of this study was to examine the associations of parental monitoring, parent–adolescent SRH communications, and parenting styles with risky sexual practices among adolescents in Harar, Ethiopia.

**Designs:**

This was a cross-sectional study conducted on adolescents aged 13–18 who had sexual initiations. Adolescents who failed to use any contraceptive method and/or condom during last sexual intercourse and who experienced multiple sexual partners in the 12 months prior to the study were taken as ‘at risk’. In view of these, the adolescents risk count ranged from zero to three – greater number indicates higher count of risky sexual practices. Poisson regression model was used to examine the associations and *p*<0.05 indicated a statistical significance.

**Results:**

It was found out that 301 of 633 (47.55%; 95% CI=43.62%, 51.45%) adolescents experienced one or more risky sexual practices. High parental monitoring compared to low decreases the Incidence Rate of engaging in risky sexual practices by 28% (adjusted incidence rate ratio, or IRR=0.72; 95% CI=0.520, 0.986). Those who had a satisfactory level of SRH communications with their parents compared to poor communicators experianced less incidence rate of risky sexual practices which was marginal (adjusted IRR=0.82; 95% CI=0.637, 1.051).

**Conclusions:**

A significant proportion of the adolescents engaged in one or more risky sexual practices. Importantly, high parental monitoring decreases the likelihood of these risky practices. Therefore, parents need to be encouraged to keep an eye on their young children.

Regardless of concerted efforts to meet universal access to sexual and reproductive health (SRH) services, various reports revealed that many adolescents were still perilously afflicted by sexually transmitted infection (STI)/HIV/AIDS, and unwanted pregnancy and associated complications (1, 2). Globally, more than 2 million young people are living with HIV and 1 in 20 contracts STI (other than HIV) each year (3, 4). Every year, about 14 million girls (aged 15–19) conceive and about 6 million of these are unintended, due to failure to use contraceptives (5–7).

These health problems are prevalent in sub-Saharan Africa (8, 9), where condoms and other contraceptive methods are hardly used and many young people experience multiple sexual partnerships. For instance, it was reported that about two thirds of the unmarried sexually active adolescents aged 15–19 in Uganda (10) and 61% of the adolescents in the age range 10–19 in Tanzania (11) did not use a condom during their last sexual intercourse. In Tanzania, 42% of the particular subjects experienced multiple sexual partnerships in the 12 months before the survey (11). Likewise, a study from west Ethiopia showed that more than half (57.3%) of the young people failed to use condoms consis'tently and about one-third of them experienced two or more lifetime sexual partners (12).

Existing findings, mainly from developed countries, have shown that high parental monitoring, SRH communication, and good parenting styles protect the adolescents’ from risky sexual practices (13–15). However, very little research in sub-Saharan Africa, including Ethiopia, has approached the problem from this perspective and the results generated from existing studies are inconclusive (12, 16, 17). In this regard, there have been calls for further research to address the SRH of young people (18, 19). Therefore, this study was done to assess the association of parental monitoring, parent–adolescent SRH communication, and parenting styles with adolescents’ risky sexual practices in Harar, Ethiopia.

## Designs

### Study setting

This study was done in Harar town, which is 510 km from Addis Ababa (the capital city of Ethiopia) to the east. The town is divided into six districts consisting of 19 kebeles (the lowest administrative unit in the country). In 2010, its population was about 100,000 people of which adolescents make up about one-fourth. About 50 ethnic groups live in the town; Oromo, Amhara, and Harari are the major ones. The town has registered more than 100% health service coverage and more than 80% educational coverage. Trade is the main livelihood of the people in Harar. Khat (*Catha edulis*) – stimulant green leaf – constitutes a significant share of the trade. Khat chewing is prevalent in the community; about a quarter of young people do it (20–22). The study was conducted from March to July 2012.

### Design and samples

The study design is cross-sectional so that all the kebeles in the town were included. Households with eligible adolescents were all considered. Adolescents aged 13–18 who resided with a parent in the town in the last 6 months and living with their parents were eligible. A house-to-house census was conducted to identify the eligible ones. When two or more eligible adolescents were found in a household, one of them was selected by a lottery method. Of the 4,559 adolescents who were interviewed, 641 who reported sexual initiation were included. Those with mental problems, unable to give assent or whose parents were unwilling to give consent, and not found at home after repeated visits (five times within a period of 1 month) were excluded.

### Data collection

Data were collected using a structured questionnaire which was first prepared in English and then translated into Amharic and Afan Oromo. Essential amendments were made to the content, language, and format of the questionnaire after it had been pre-tested and piloted. The instrument had different parts: the first part addressed the socio-demographic characteristics of the respondents and then adolescent and parent relationships, parental monitoring, parenting styles, and SRH communications. Questions related to sexuality were put at the middle of the tool. Less sensitive questions were asked before the sensitive ones, as per recommendations to design a tool for sexual survey (23–25). The less sensitive sexual milestone questions (menstruation for female and wet dream for male adolescents and then questions of early romantic relationships ever experienced, i.e., girlfriend or boyfriend, and any practice of kissing and male/female genital touching) were asked first. Respondents were then asked about ever experiencing sexual intercourse, using any contraceptive methods and condoms at last sexual intercourse, and about the number of sexual partners overall and also in the 12 months prior to the interview. The data collection technique was a face-to-face interview. Male respondents were interviewed by male data collectors while female respondents were interviewed by female data collectors. The data collectors were thoroughly trained on the questionnaire and data gathering procedures. They were also trained on how to establish rapport and conduct the interview with the correct approaches. The data collection activities were supervised daily by trained supervisors and closely monitored by the principal investigator.

### Measurements

The dependent variable was the risky sexual practices of the study subjects. This was defined as not using a condom and/or contraceptive method during their last sexual intercourse, and having multiple sexual partners in the 12 months prior to the interview. Adolescents who never used any contraceptive method (modern) had two risk counts – risks for pregnancy and STI/HIV/AIDS. Those who used other contraceptive methods but did not use a condom had one risk count – risk for STI/HIV/AIDS, and those who had experienced more than one sexual partner in 12 months prior to the study had one risk count. In general, the respondents had sexual risk score counts which ranged from zero to three represented as minimal risk (zero), moderate risk (one), and high risk (two) and very high risk (three). Similar sexual risk assessments had been used (26, 27).

The main independent variables were parental monitoring, SRH communication, and parenting styles which are also called parenting practices. The adolescents maternal/paternal monitoring was assessed using a scale of five items adapted from past studies (28, 29). These items measured how often the mother/father know what he/she does in his/her free time; where he/she goes at night; who his/her friends are; how he/she spends his/her money; and where he/she goes after school. Responses ranged from ‘not at all’ (coded as 1) to ‘always’ (coded as 5). The scale had good internal consistency of Cronbach α greater than 0.71. Then, 33rd and 67th percentiles of the composite scores of both maternal and paternal monitoring were used to categorize them into low (<33rd percentile), medium (between 33rd and 67th percentiles; inclusive), and high (>67th percentile). When there were discordances from the maternal or paternal monitoring, the higher one of the two was taken to create one parental monitoring variable. For example, when there was high maternal parenting and low paternal monitoring, the high maternal monitoring was taken.

The SRH communication between mother–adolescent and father–adolescent was assessed using a nine-itemed scale adapted from past studies (30, 31), which asked how often the adolescents and mother/father communicate about choosing a boyfriend or girlfriend, birth control, condoms, HIV/AIDS, reproduction/contraceptive, reproductive organ growth and development, STI (other than HIV/AIDS), and when to start sex. Responses ranged from ‘not at all’ (coded as 1) to ‘always’ (coded as 5). The scale had good internal consistency of Cronbach α greater than 0.81. Then, 33rd and 67th percentiles composite score of mother/father–adolescent SRH communication was used to categorize the communication level into very poor communicators (<33rd percentile), poor communicators (between 33rd and 67th percentiles; inclusive), and satisfactory communicators (>67th percentile). Similar approaches that had been presented under the parental monitoring were applied in order to create a variable for further analysis.

The parenting styles were assessed using two questions which looked into the adolescents’ perception of their mother/father supportiveness (very supportive or not very/somewhat supportive) and strictness (strict or permissive) (32, 33). The questions included how the mother/father is supportive to the adolescent (indicate responsiveness) and how the mother/father is strict in making sure that what the adolescent needs to do is done (indicate demandingness). These responses were combined to create four parenting styles that include ‘neglectful’ (permissive and not very/somewhat supportive), ‘permissive’ (permissive and very supportive), ‘authoritarian’ (strict and not very/somewhat supportive), and ‘authoritative’ (strict and very supportive). When the adolescents reported the mother and the father exhibiting different parenting styles: authoritative than (the authoritarian, permissive and neglectful); authoritarian than (permissive and neglectful); and permissive than neglectful were taken to create a parenting style variable that the adolescents experianced.

Other variables were the socio-demographic and economic characteristics of the respondents. These included subject age categorized into age groups 13–14, 15–16, and 17–18 years; sex of the adolescent was coded as male and female; educational status of adolescents was categorized into primary and less and secondary and more; family wealth index classified into low, medium, and high and data not obtained; adolescents living arrangements was classified as living with both the biological mother and father and other living arrangements. Monthly expendable pocket money was categorized in to less than or equal to 100 Ethiopian birr (ETB), 101–200 ETB, and more than 200 ETB (1$=19.59 ETB). Adolescents’ alcohol consumption and chewing Khat were categorized into yes/no.

### Statistical analysis

The data were double entered and cleaned using EpiData software (Version 3.1), and then exported to STATA (Version 12) for analysis. Poisson regression was applied to investigate the associations of the independent variables with the outcome variable. Poisson regression is a modeling technique used in order to analyze count outcome variables. The model handles the outcome count changes in relation to the independent variables (34). Bivariate analysis was first made and the variables with a *p*-value less than 0.25 were considered for subsequent model building. Important variables deemed to be considered were also included, though they did not reach a *p*-value less than 0.25. Model-I was built to examine the association of parenting practices alone. Subsequently, relevant socio-demographic variables were included (Model-II) to assess the associations of the parenting practices when controlled for these variables. The final model was built including all the variables in Model-I, Model-II and adolescent behavior-related variables (alcohol drinking and Khat chewing). Multicollinearity was assessed by variance inflation factor (VIF) and interactions were also checked among the independent variables. The results were considered statistically significant at *p*<0.05.

### Ethical statement

Ethical approval was obtained from the Institutional Research Ethics Review Committee of the Colleges of Health and Medical Sciences of Haramaya University. Informed consent and ascent statements were presented on the first page of the data collection tool. Written consent was obtained from the parents and written assent was secured from the adolescents after explaining the objective, design, and the implication of the study. The interviews were conducted in private and all information used in this study was handled with anonymity. Each respondent was clearly explained that participation is voluntary and that they can decline at any time without any explanation if they wish to do so.

## Results

Of the 641 adolescents who reported sexual initiation, eight respondents were excluded from the analysis because their responses on the outcome variable were incomplete. The final analysis included 633 adolescents who had complete reports. From these, the majority (71.88%) was aged 17–18 and 80.73% were in-school adolescents. A total of 268 (41.71%) were living with their biological mother and father. A few reported high parental monitoring (16.59%) while 47.08% of them reported an authoritative parenting style. About half of them (51.82%) had a satisfactory level of communications on SRH with the parent (Table 1).

**Table 1:** Socio-demographic characteristics and parenting practices of adolescents in Harar, Ethiopia, 2012

Variables/characteristics	Number (*N*=633)	%
Sex of the adolescents		
Male	306	48.34
Female	327	51.66
Age of the adolescents (years)		
13–14	24	3.79
15–16	154	24.33
17–18	455	71.88
Adolescent educational status		
Grade eight and less	216	34.12
Grade nine and more	417	65.88
Schooling of the adolescents		
In-school	511	80.73
Out-of-school	122	19.27
Adolescent expendable pocket money per month		
≤100 ETB	299	47.24
101–200 ETB	153	24.17
More than 200 ETB	181	28.59
Adolescents living arrangements		
With both biological mother and father	264	41.71
With other living arrangements^a^	369	58.29
Parental monitoring		
Low	315	49.76
Medium	213	33.65
High	105	16.59
SRH communication level		
Very poor communication	150	23.70
Poor communication	155	24.49
Satisfactory communication	328	51.82
Parenting styles		
Neglectful	82	12.95
Permissive	162	25.59
Authoritarian	91	14.38
Authoritative	298	47.08

a Other living arrangement included living with mother alone/fatheralone and the parent figure head/the guardian. ETB=Ethiopian birr; SRH=sexual and reproductive health communication.

From the 633 respondents, 72 (11.37%) did not use any contraceptive method and 30.7% did not use a condom during their last sexual intercourse, and 21.38% reported multiple sexual partners 12 months prior to the study. Overall, 301 of 633 (47.55%; 95% CI=43.62%, 51.45%) adolescents engaged in one or more risky sexual practices (Table 2).

**Table 2:** Risky sexual practices of adolescents in Harar, Ethiopia, 2012

Risky sexual practices	Total (*N*=633)	Female (*N*=327)	Male (*N*=306)
Contraceptive use (any) during last sexual intercourse			
No	72 (11.37)	43 (13.15)	29 (9.48)
Yes	561 (88.63)	284 (86.85)	277 (90.52)
Condom use during last sexual intercourse^a^			
No	195 (30.81)	142 (43.43)	53 (17.32)
Yes	438 (69.19)	185 (56.57)	253 (82.68)
Multiple sexual partnership in last 12 months			
No	498 (78.67)	289 (88.38)	208 (67.98)
Yes	135 (21.33)	38 (11.62)	98 (32.02)
Sexual risky practices			
Minimal risk (zero)	332 (52.45)	151 (48.62)	171 (56.58)
Moderate risk (one)	216 (34.12)	120 (36.62)	96 (31.25)
High risk (two)	75 (11.92)	45 (13.85)	30 (9.87)
Very high risk (three)	10 (1.59)	3 (0.92)	7 (2.30)

a Is to mean male condoms.

High parental monitoring compared to low monitoring decrease the incidence rate of risky sexual practices by 28% (adjusted incidence rate ratio, or IRR=0.72; 95% CI=0.520, 0.981). The adolescents who had a satisfactory level of parent–adolescent communication on sexual and reproductive matter compared to those who had very poor communication experianced less incidence rate of risky sexual practices (adjusted IRR=0.82; 95% CI=0.637, 1.051) – marginal. There was no association between parenting style and the adolescents’ risky sexual practices. From the rest of the variables modeled together, adolescents who had ever chewed Khat were more likely to have experienced a higher incidence rate risky sexual practices (adjusted IRR=1.33; 95% CI=1.065, 1.659) (Table 3).

**Table 3:** Multivariable Poisson regression of factor associated with adolescents engagements in risky sexual practices, in Harar, Ethiopia, 2012

	Risky sexual practices			
	
	Bivariate model	Model-I	Model-II	Final model
	
Variables/characteristics	Crude IRR (95% CI)	Adj. IRR (95% CI)	Adj. IRR (95% CI)	Adj. IRR (95% CI)
Parental monitoring				
Low	1 (Ref)	1 (Ref)	1 (Ref)	1 (Ref)
Medium	0.84 (0.676, 1.048)	0.71 (0.705, 1.102)	0.89 (0.717, 1.128)	0.92 (0.729, 1.148)
High	0.65 (0.479, 0.895)^a^	0.88 (0.515, 0.974)^a^	0.70 (0.508, 0.971)^a^	0.72 (0.520, 0.986)^a^
SRH communication				
Very poor	1 (Ref)	1 (Ref)	1 (Ref)	1 (Ref)
Poor	0.84 (0.646, 1.107)	0.90 (0.687, 1.192)	0.92 (0.699, 1.221)	0.94 (0.723, 1.271)
Satisfactory	0.73 (0.579, 0.926)^a^	0.80 (0.629, 1.025)	0.79 (0.622, 1.025)	0.82 (0.637, 1.051)
Parenting styles				
Neglectful	1 (Ref)	1 (Ref)	1 (Ref)	1 (Ref)
Permissive	0.92 (0.674, 1.256)	0.92 (0.674, 1.257)	0.96 (0.703, 1.331)	0.94 (0.685, 1.300)
Authoritarian	0.82 (0.572, 1.178)	0.90 (0.625, 1.300)	1.00 (0.688, 1.464)	1.00 (0.686, 1.471)
Authoritative	0.71 (0.536, 0.965)^a^	0.77 (0.571, 1.039)	0.86 (0.627, 1.178)	0.85 (0.619, 1.168)
Sex of the adolescent				
Male	1 (Ref)		1 (Ref)	1 (Ref)
Female	1.16 (0.949, 1.413)		1.19 (0.963, 1.490)	1.23 (0.985, 1.537)
Adolescent living arrangements				
With biological mother and father	1 (Ref)		1 (Ref)	1 (Ref)
Other living arrangements	1.09 (0.898, 1.344)		1.01 (0.819, 1.262)	1.03 (0.825, 1.272)
Family wealth index				
Low	1 (Ref)		1 (Ref)	1 (Ref)
Medium	1.16 (0.901, 1.482)		1.11 (0.862, 1.426)	1.11 (0.859, 1.423)
High	0.78 (0.591, 1.056)		0.81 (0.598, 1.084)	0.82 (0.607, 1.100)
Data-not obtained	0.95 (0.702, 1.283)		0.89 (0.657, 1.228)	0.89 (0.649, 1.214)
Expendable pocket money per month				
≤100 ETB	1 (Ref)		1 (Ref)	1 (Ref)
101–200 ETB	0.92 (0.716, 1.190)		0.92 (0.714, 1.197)	0.88 (0.676, 1.140)
Greater than 200 ETB	1.11 (0.881, 1.390)		1.09 (0.861, 1.403)	1.05 (0.817, 1.346)
Schooling of the adolescents				
In-school	1 (Ref)		1 (Ref)	1 (Ref)
Out-of-school	1.39 (1.111, 1.751)^a^		1.23 (0.953, 1.576)	1.23 (0.958, 1.591)
Ever drunk alcohol				
No	1 (Ref)			1 (Ref)
Yes	0.93 (0.765, 1.136)			0.89 (0.713, 1.106)
Ever chewed Khat				
No	1 (Ref)			1 (Ref)
Yes	1.26 (1.035, 1.537)^a^			1.33 (1.065, 1.659)^a^

a Significant at *p*<0.05. IRR=incidence rate ratio; SRH=sexual and reproductive health communication; ETB=Ethiopian birr.

## Discussion

This community-based study revealed that 47.55% of the adolescents experienced one or more risky sexual practices. About one-third did not use a condom at their last sexual intercourse and one in five had a history of multiple sexual partnerships in 1 year prior to the interview. Those who had been experiencing high parental monitoring were less likely to engage in higher numbers of risky sexual practices. Adolescents who had satisfactory levels of sexual reproductive communication were less likely to report a higher number of risky sexual practices – the significance was marginal. There was no significant association between the parenting styles and the sexual risk practices. Chewing Khat was significantly associated with a higher number of risky sexual practices.

About one-third of the adolescents did not use condom during their last sexual intercourse. This finding is similar with a result reported by another study from Ethiopia (12). The reasons for not using condoms are largely related to social and psychological factors (35). The social norm in Ethiopia, as elsewhere, discourages premarital sex, and this makes adolescents feel uncomfortable to buy and possess condoms. Moreover, the pleasure concern might also explain the finding (36). However, the unprotected sexual intercourse prevalence in our study is less than those findings reported from sub-Saharan African regions (11, 18) but higher than other report from Cape Verde, West Africa (37). These discrepancies might be linked with variablities in service availability, accessibility, and demographic characteristics.

One-fifth of the adolescents had experienced multiple sexual partners in 1 year prior to the interview. Many reasons can be posited for why to engage in risky sexual practices which are likely linked with economic/social challenges/influences, especially for the female, and the masculinity and sexual prestige for male adolescents (38). One probable explanation, though it needs to be explored further, in this study setting is chewing Khat. This is a prevalent practice, and ritualistically it is common that both males and females chew Khat together. This condition might allow them to have more interactions, and it has been known that interactions in a frequent manner habitually create a sense of intimacy that might eventually result in romantic and/or sexual relationships.

Parental monitoring decreased the likelihood of engaging in a higher number of sexual risky practices. This result further built on previous findings from Ethiopia (12) and Tanzania (18). This might indicate that adolescents potentially act responsibly against risky sexual practices when there is high parental monitoring due to the fact that parental monitoring enhances self-esteem of the young people (39). Moreover, parental monitoring can indirectly protect them from other risky behaviors which have a potentiating effect on the adolescents’ risky sexual practices (15). Overall, this illuminates that parental monitoring may be an important perspective to be considered in an effort to improve the adolescents’ SRH.

Adolescents who had a satisfactory level of SRH communication with their parents were less engaged in risky sexual practices, though the significance level was marginal. A similar pattern of association has been found in a previous study (35). This might be explained along with the existing theory that repetitive communications would facilitate better learning and retention of messages (40). This could help the adolescents to cope with various sexual demands that arise both from internal desire as well external pressure (41, 42). The marginality level of significance might be because in some cases SRH communications may not be intended for teaching but rather a negative reaction and take the form of frustration (43).

No significant associations observed between the parenting styles and risky sexual practices. The parenting styles might have taken an indirect path, which was not considered in this study. This indicate the needs to further explore the different paths through which the parenting styles could have an effect on the adolescents’ risky sexual practices.

Chewing Khat was associated with engagement in a higher number of risky sexual practices, and this is consistent with a previous study (44). Khat is a stimulant substance which contains amphetamines and excites the individuals’ functional activity. It is also linked to the consumption of other substances which might further trigger engagement in risky sexual practices.

## Limitations

This study has some limitations that need be reported, despite efforts made to control/minimize them. Although the questionnaire was framed based on exiting recommendations, loading questioning techniques was applied, interviews were conducted in private places to ensure the privacy, data collectors were thoroughly trained, gender-match was made between the data collectors and respondents, and also we considered interviewers age range preferred by adolescents, there might be over- or under-reporting due to social desirability bias and the face-to-face interview method. Because the study was done in an urban setting, the findings may not be inferred to semi-urban and rural contexts. Moreover, causality may not be presumed due to the cross-sectional nature of the study design.

## Conclusions

In this study, a significant proportion of adolescents engaged in risky sexual activities. These risky sexual practices would expose them to various short- and long-term health issues like unwanted pregnancy, STI/HIV/AIDS, psychological, and social health problems. Importantly, high parental monitoring decreases the likelihood of engaging in higher numbers of sexual encounters. SRH communications made between parents and adolescents play considerable roles in minimizing these risks. Enabling the parent to exhibit high parental monitoring and enhancing SRH communications between parent and the adolescents is imperative. These can be done through various modalities of interventions at home, in school, and in the work place.
